# A Delphi Technology Foresight Study: Mapping Social Construction of Scientific Evidence on Metagenomics Tests for Water Safety

**DOI:** 10.1371/journal.pone.0129706

**Published:** 2015-06-11

**Authors:** Stanislav Birko, Edward S. Dove, Vural Özdemir

**Affiliations:** 1 Faculty of Communications and the Department of Industrial Engineering, Office of the President, International Technology and Innovation Policy, Gaziantep University, Üniversite Bulvarı, Şehitkamil, Gaziantep, 27310, Turkey; 2 School of Biotechnology, Amrita Vishwa Vidyapeetham (Amrita University), Kerala, India; 3 Centre of Genomics and Policy, Department of Human Genetics, Faculty of Medicine, McGill University, Montreal, QC, Canada; 4 J. Kenyon Mason Institute for Medicine, Life Sciences and the Law, University of Edinburgh School of Law, Edinburgh, United Kingdom; Emory University, UNITED STATES

## Abstract

Access to clean water is a grand challenge in the 21^st^ century. Water safety testing for pathogens currently depends on surrogate measures such as fecal indicator bacteria (e.g., *E*. *coli*). Metagenomics concerns high-throughput, culture-independent, unbiased shotgun sequencing of DNA from environmental samples that might transform water safety by detecting waterborne pathogens directly instead of their surrogates. Yet emerging innovations such as metagenomics are often fiercely contested. Innovations are subject to shaping/construction not only by technology but also social systems/values in which they are embedded, such as experts’ attitudes towards new scientific evidence. We conducted a classic three-round Delphi survey, comprised of 107 questions. A multidisciplinary expert panel (n = 24) representing the continuum of discovery scientists and policymakers evaluated the emergence of metagenomics tests. To the best of our knowledge, we report here the first Delphi foresight study of experts’ attitudes on (1) the top 10 priority evidentiary criteria for adoption of metagenomics tests for water safety, (2) the specific issues critical to governance of metagenomics innovation trajectory where there is consensus or dissensus among experts, (3) the anticipated time lapse from discovery to practice of metagenomics tests, and (4) the role and timing of public engagement in development of metagenomics tests. The ability of a test to distinguish between harmful and benign waterborne organisms, analytical/clinical sensitivity, and reproducibility were the top three evidentiary criteria for adoption of metagenomics. Experts agree that metagenomic testing will provide novel information but there is dissensus on whether metagenomics will replace the current water safety testing methods or impact the public health end points (e.g., reduction in boil water advisories). Interestingly, experts view the publics relevant in a “downstream capacity” for adoption of metagenomics rather than a co-productionist role at the “upstream” scientific design stage of metagenomics tests. In summary, these findings offer strategic foresight to govern metagenomics innovations *symmetrically*: by identifying areas where acceleration (e.g., consensus areas) and deceleration/reconsideration (e.g., dissensus areas) of the innovation trajectory might be warranted. Additionally, we show how scientific evidence is subject to potential social construction by experts’ value systems and the need for greater upstream public engagement on metagenomics innovations.

## Introduction

Water scarcity in the face of an exponentially growing world population and management of safe water are grand challenges for 21^st^ century science and society [1, 2]. The Millennium Ecosystem Assessment Report led by a consortium of more than 1360 experts and called for in 2000 by Kofi Annan, the then Secretary-General of the United Nations, indicated that the aquatic environment will not be able to sustain anticipated population growth. Worrisomely, the report cogently expressed that:

Over the past 50 years, humans have changed ecosystems more rapidly and extensively than in any comparable period of time in human history, largely to meet rapidly growing demands for food, fresh water, timber, fiber and fuel. This has resulted in a substantial and largely irreversible loss in the diversity of life on Earth [3].

Moving forward in the current era of the United Nations Sustainable Development Goals (SDGs), due to be in effect from 2016 to 2030, water safety testing is no doubt a crucial foundation for ecosystem health. The water quality monitoring presently depends on surrogate measures such as fecal indicator bacteria (e.g., *E*. *coli*) grown in culture to predict the risk of waterborne pathogens. However, a vast majority of microorganisms– 99%–cannot be grown readily in culture, and not all fecal indicators correlate well with pathogens [4–6].

Historically, the often cited “great plate count anomaly” has provided the early hints that the cultured microorganisms did not represent much of the microbial world. This was evidenced by the discrepancy between the sizes of populations estimated by dilution plating and by microscopy [7, 8]. In some aquatic environmental samples, plate counts and viable cells estimated by acridine orange staining differ by four to six orders of magnitude [9].

Not surprisingly, studies in the past failed to establish a robust link between occurrence of pathogens and cultured indicator organisms such as *E*. *coli* [10]. Also, waterborne outbreaks have occurred in the absence of positive indicator test results [11]. A recent analysis conducted in the Upper Mississippi River in Minnesota showed the concentration of *E*. *coli*, used as an indicator of surface water quality and human health risk by the United States Environmental Protection Agency (EPA), was not correlated with other measures of water quality such as nitrogen concentration or abundances of certain human pathogens (e.g., *Enterobacteriales*) [12].

The molecular methods in water safety monitoring are not new. Quantitative polymerase chain reaction as an alternative to the culture-based indicators of water quality has existed for some time [8]. On the other hand, omics biotechnologies (e.g., genomics, proteomics and metagenomics) offer a system-wide unbiased survey of biological systems and pathophysiological medical and ecosystem conditions [13]. Metagenomics is a rapidly emerging omics biotechnology that has promise as a “game changer” to transform water safety by detecting the pathogens directly, instead of their surrogates. Metagenomics concerns the high-throughput, culture-independent, unbiased shotgun sequencing of DNA from environmental samples [14–17]. The word metagenomics was coined [18] building on the idea of cloning DNA directly from environmental samples, first noted by Pace [19]. Importantly, we underscore the difference between metagenomics (environmental shotgun sequencing) and 16S amplicon sequencing. The latter is far more commonly used for species enumeration at the current time and often confused in the literature as metagenomics. Recent water safety research has focused to sample the metagenome (the combined genomes of the organisms present) to measure changes in microbial communities associated with chemical and other environmental perturbations [12]. Metagenomics studies, on the other hand, examine in the spirit of omics systems sciences the totality of genetic material recovered directly from a sample rather than a specific gene or the genome of a single organism. Metagenomics permits the study of microbial communities in their natural habitats; the findings can include taxonomic profiles (which families, genera, or species are present in a community?) or functional profiles (which sets of genes are community members expressing in relation to the community’s niche in the ecosystem?) [15–17]. Metagenomics discovery platforms can also pave the way for PCR-based analyses of a targeted set of biomarkers associated with pathogens in environmental samples.

Prior to introduction of metagenomics approaches, the full diversity of microbial communities that co-exist with human populations was virtually unknown. Metagenomics offers the potential for a host of radical advances in postgenomics microbiology, environmental sciences, water safety testing and characterization of pathogens that have hitherto escaped detection by conventional culture-based diagnostic methods, and a deeper understanding of the host-ecosystem interactions [20–23].

Despite the promises of metagenomics biotechnology for public health and ecosystem (e.g., water) safety, lessons from the field of new technology governance underscore that the sought after beneficial outcomes of novel biotechnologies do *not* automatically flow from a marriage of biology and technology, nor does the development of technological capability suffice in ensuring responsible innovation that is grounded in societal values and priorities [24–29].

**Fig 1:**
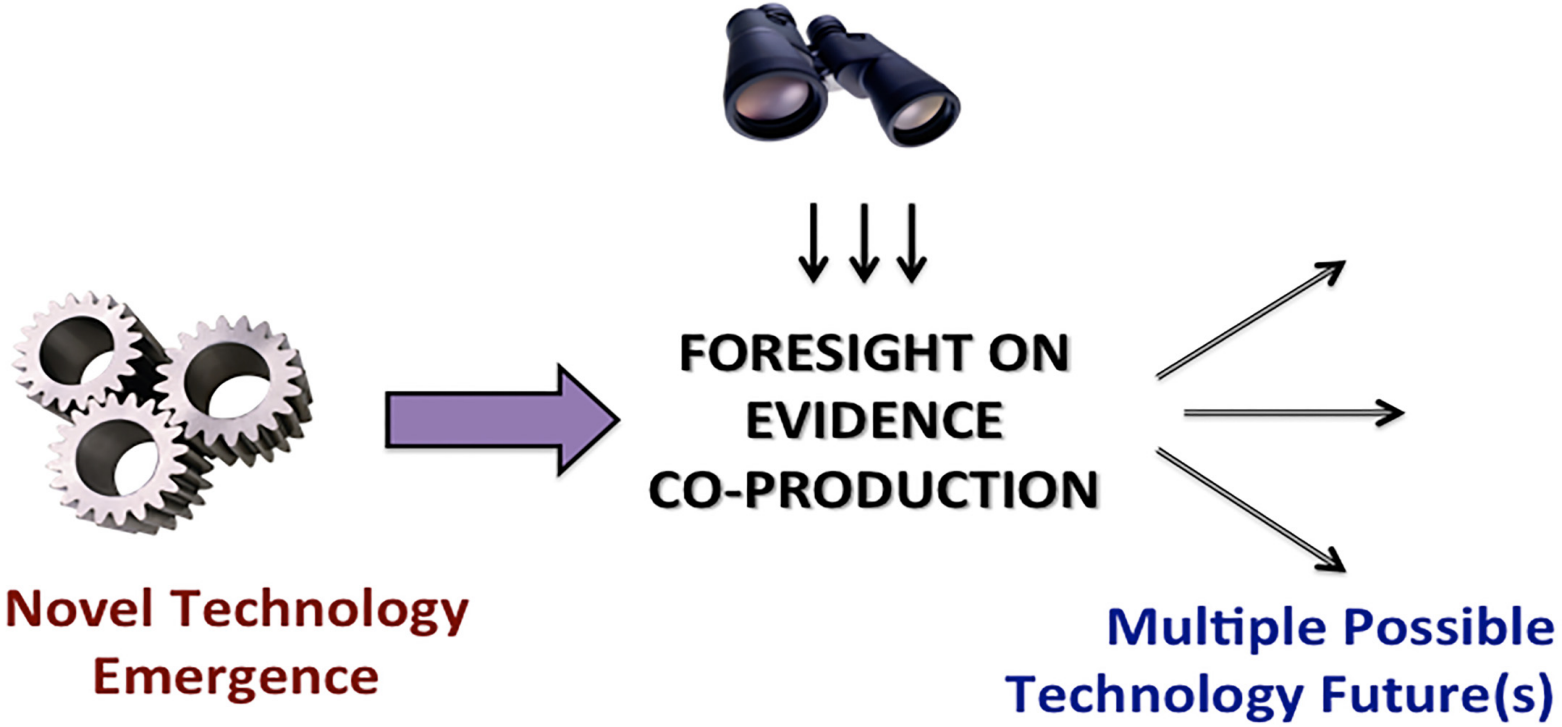
Evidence as the focus of foresight research. Situated conceptually between novel technology *emergence* and *adoption future(s)*, examination of the attitudes towards co-production of technology-related evidence by the innovation ecosystem constituents (e.g., scientists and policymakers) can help build strategic foresight on the innovation trajectory.

Foresight research examines the multiple possible ways in which biotechnology innovation future(s) might materialize [30]. While scenario planning has been a mainstay practice in technology governance and foresight, the production of scientific evidence as a *precursor* to adoption of technology discoveries, and the ways in which the concept of evidence may be perceived and prioritized by stakeholders, have been little examined to date. In other words, understanding how scientific evidence is co-produced in divergent or convergent ways by the constituents of an innovation ecosystem is instrumental in understanding the possible innovation futures for the metagenomics technology (**Fig 1**).

With the above overarching context and rationale, we report, to the best of our knowledge, the first Delphi study on the emergence of metagenomics tests for water safety, in regards to science and policy experts’ attitudes towards:

1. the top 10 priority evidentiary decision criteria for adoption of metagenomics tests,
2. the specific issues pertinent to governance of the metagenomics innovation trajectory where there is consensus or dissensus among experts,
3. the anticipated time lapse from discovery to practice of metagenomics tests, and
4. the role and timing of public engagement in development of metagenomics tests.

This classic three-round Delphi study sought answers to the above pressing questions in 21^st^ century life sciences broadly, and water safety testing and ecosystem health specifically. We surveyed the experts in regards to their values and attitudes on the entire innovation trajectory driven by metagenomics discovery platform, with a view to anticipated future emergence of novel molecular diagnostics for detection of specific waterborne pathogens. To the best of our knowledge there are presently no routine water safety tests with regulatory approval in public health practice that are developed by metagenomics discovery approaches but commercially available tests can be anticipated in the near future. Moving forward, the technology governance literature uniformly underscores the importance of foresight research at an early upstream (design) stage when a new technology is still flexible and emerging rather than as a hindsight after a technology is entrenched and when it is very difficult to shape or steer firm beliefs on established innovation [28–32].

The Delphi findings reported here illustrate the experts’ consensus (and the knowledge domains where consensus is lacking) towards evidence perceived to be essential to move metagenomics discoveries to metagenomics tests. Collectively, this foresight research informs concrete new strategies to devise future roadmaps to responsibly steer metagenomics laboratory discoveries to new tests for water safety, and may also help for rational technology transfer and commercialization in the near future.

## Materials and Methods

### Study Design and Process Flowchart

**Fig 2:**
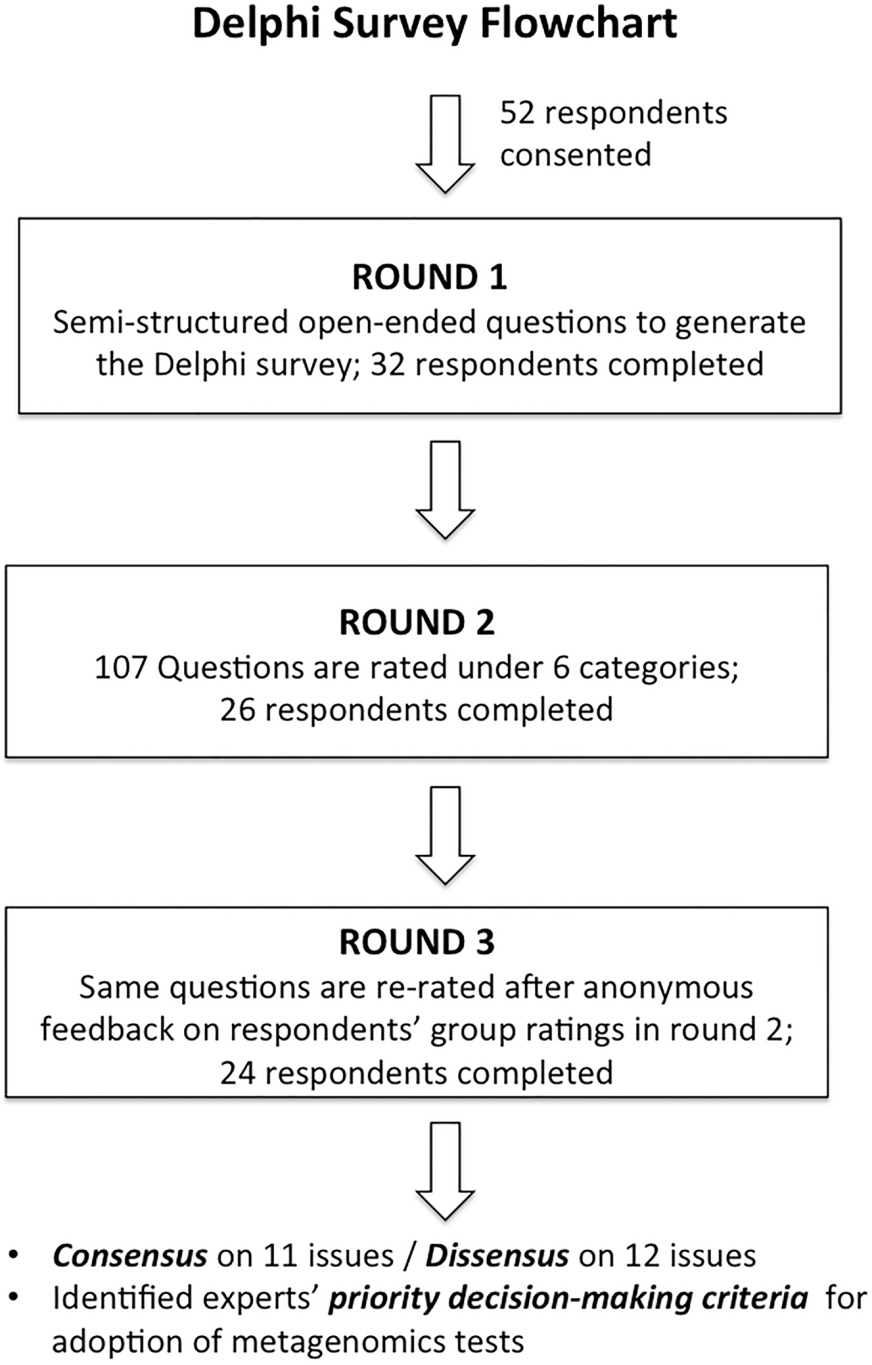
Delphi study flowchart.

We conducted a Delphi survey to examine the issues posed in the introduction. The Delphi process followed is summarized in **Fig 2**. The study was conducted from 2013 to 2014 at McGill University, Canada. Written informed consent was obtained from all participants by fax and e-mail. Ethics approval was obtained through the Faculty of Medicine at McGill University (Institutional Review Board Assurance # FWA 00004545). Routine communications with panelists were conducted by e-mail.

The Delphi survey is a group communication technique based on an interactive, sequential and multi-step characterization of expert stakeholders, their interests, and intersection of interests. It has the advantage of obtaining opinion from experts, with a guarantee of anonymity, avoiding the potential distortion caused by peer pressure in group situations such as focus group analysis.

The classic Delphi study has three rounds: (1) a general questionnaire asking panel members to identify the pressing issues in a given knowledge domain (e.g., an emerging technology such as metagenomics); 2) a second-round questionnaire asking panel members to rate the importance of the list of the issues identified from the first round; (3) a third-round questionnaire, asking panel members to re-evaluate their ratings of each survey item after reviewing the expert panel’s collective stance in the second round in response to the survey questions [33–35].

### Respondents in the Survey

The respondents were experts spanning the science-policy spectrum relating to water safety testing with metagenomics technology. The survey questions and communications with the respondents were conducted in English. The respondent experts had, as a common denominator, a primary institutional affiliation in Canada and a track record of publications and/or professional scholarly engagements in water safety and/or omic technologies as evidenced by their curriculum vitae.

Purposive sampling followed by snowball recruitment was used for experts’ participation in the study. Fifty-two experts consented to participate in the study. Each expert was assigned a randomly generated code (between 0 and 999) by a study coordinator to safeguard anonymity. We note that the literature on Delphi surveys traditionally recommends a panel of 10 to 15 experts [35, 36], a sample size that was exceeded in the present Delphi study with a final sample of 24 experts.

### Delphi Survey

The first round Delphi survey consisted of 10 open-ended questions grouped into three themes: perceived evidentiary requirements on adoption and performance metagenomics tests, benefits and risks, and perceptions on the role of public engagement for the future development of the metagenomics innovation trajectory. This classic Delphi approach was used so as to solicit experts’ opinions on which the survey questions were generated for the remaining two rounds of the Delphi study.

**Fig 3:**
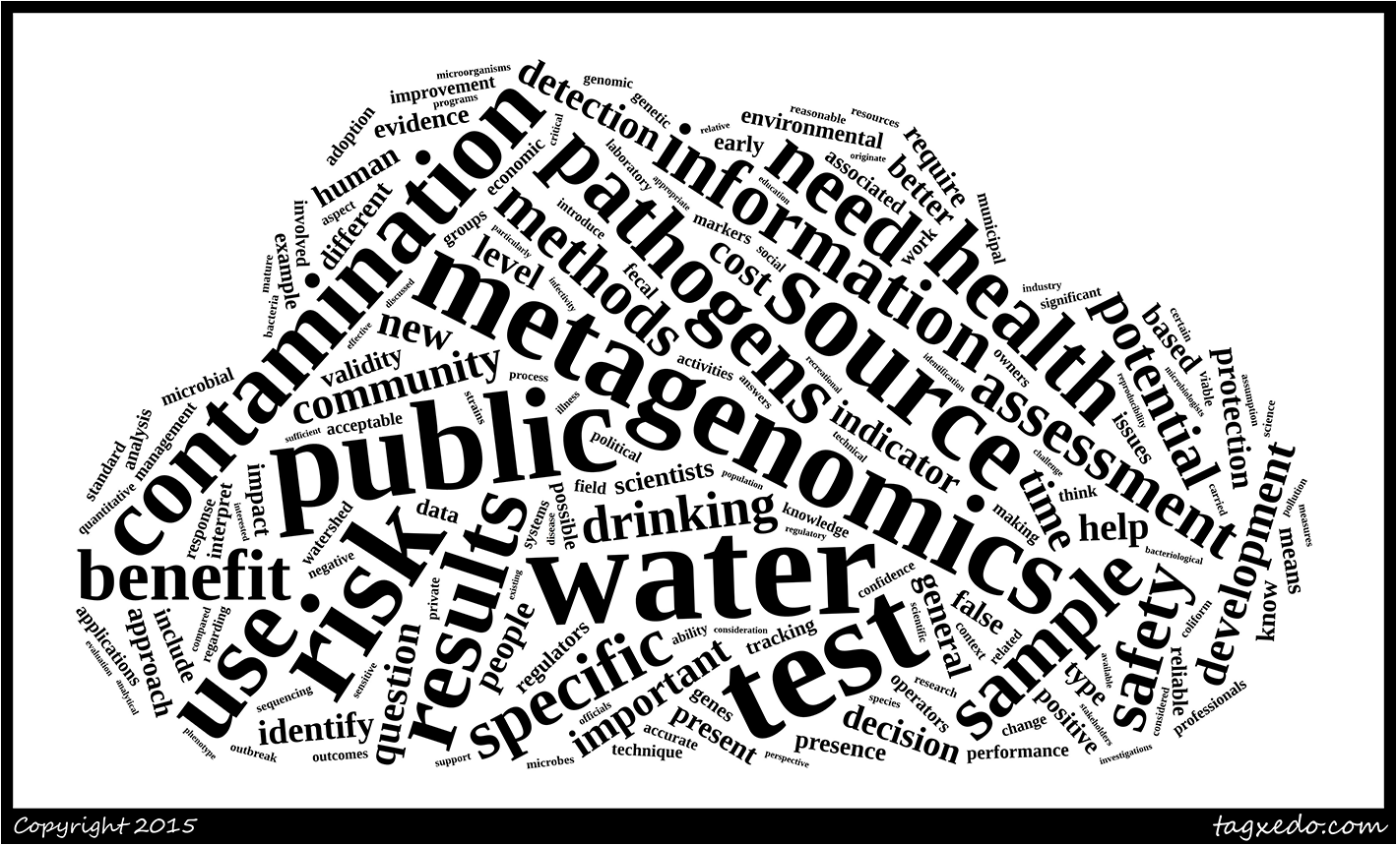
Word cloud generated from the Delphi round 1 responses.

In brief, the above first round responses were analyzed using the NVivo [37] qualitative data analysis software. A word cloud was created using the open access word cloud generator wordle.net. The latter algorithm assigns greater prominence (using font size) to words that appear more frequently in the source text (**Fig 3**). We noted the broad themes emergent from the experts’ responses and subsequently categorized the issues thematically. Repetitive headings were removed and an initial list was created. The responses were then re-read alongside the list of categories and coded accordingly. The coded sections were organized under the headings. Headings that were coded most were selected. The wording for each selected heading was chosen to represent as closely as possible the range of corresponding coded sections.

**Table 1**provides the six thematic heading categories used to generate and structure the 107 Delphi survey questions for the rounds 2 and 3. The Delphi survey questions are displayed in the **S1 Text**. The survey also included a question on the top 10 evidentiary priorities to be considered in the decision whether or not metagenomic testing should be widely adopted for water safety.

**Table 1:** Six Thematic Categories Used to Generate and Structure the Delphi Survey.

**1. Introduction and Overarching Contextual Questions**
**2. Evidentiary Requirements for Adoption of Metagenomics Tests:** Analytical validity (i.e., performance to detect a metagenomics profile); Clinical/ecosystem validity (i.e., performance of the association analyses between metagenomics profiles and the putative pathogens in water); Utility of metagenomics tests for water safety
**3. Other Attributes/Caveats of Metagenomics Tests for Water Safety:** Technical and temporal aspects (e.g., projected adoption timelines), Interpretation of tests
**4. Consequences of Metagenomics Tests for Water Safety:** Identification of pathogens, source of contamination; Understanding of the consequences; Treatment of water, standards & regulations on water safety; Economic and political consequences
**5. Timing and Context of Public Engagement for Metagenomics Tests on Water Safety:** (e.g., *when public engagement ought to happen*?*–upstream at discovery stage*, or *downstream after the tests are already developed*)
**6. Priority Issues/Criteria for Decisions on Adoption of Metagenomics Tests**

The second round survey asked the respondents to rate each question on a 7-point *agreement*, *desirability* and/or *feasibility* Likert scale (**S1 Text**), with 1 and 7 signifying the least and the most desirable/feasible, respectively [38]. Some questions were rated on more than one scale (e.g., desirability and feasibility) each of which counted as an itemized response to that question (**S1 Text**). Following the rating scales for each item, space was provided where experts were encouraged to make explicit the assumptions their ratings were based on, as well as to add any other free text comments.

The third round survey consisted of the same questions as in the second round and provided the respondents the opportunity to revise or recalibrate their ratings after having seen the group’s anonymous summary ratings from the second round.

### Data Analysis

**Fig 4:**
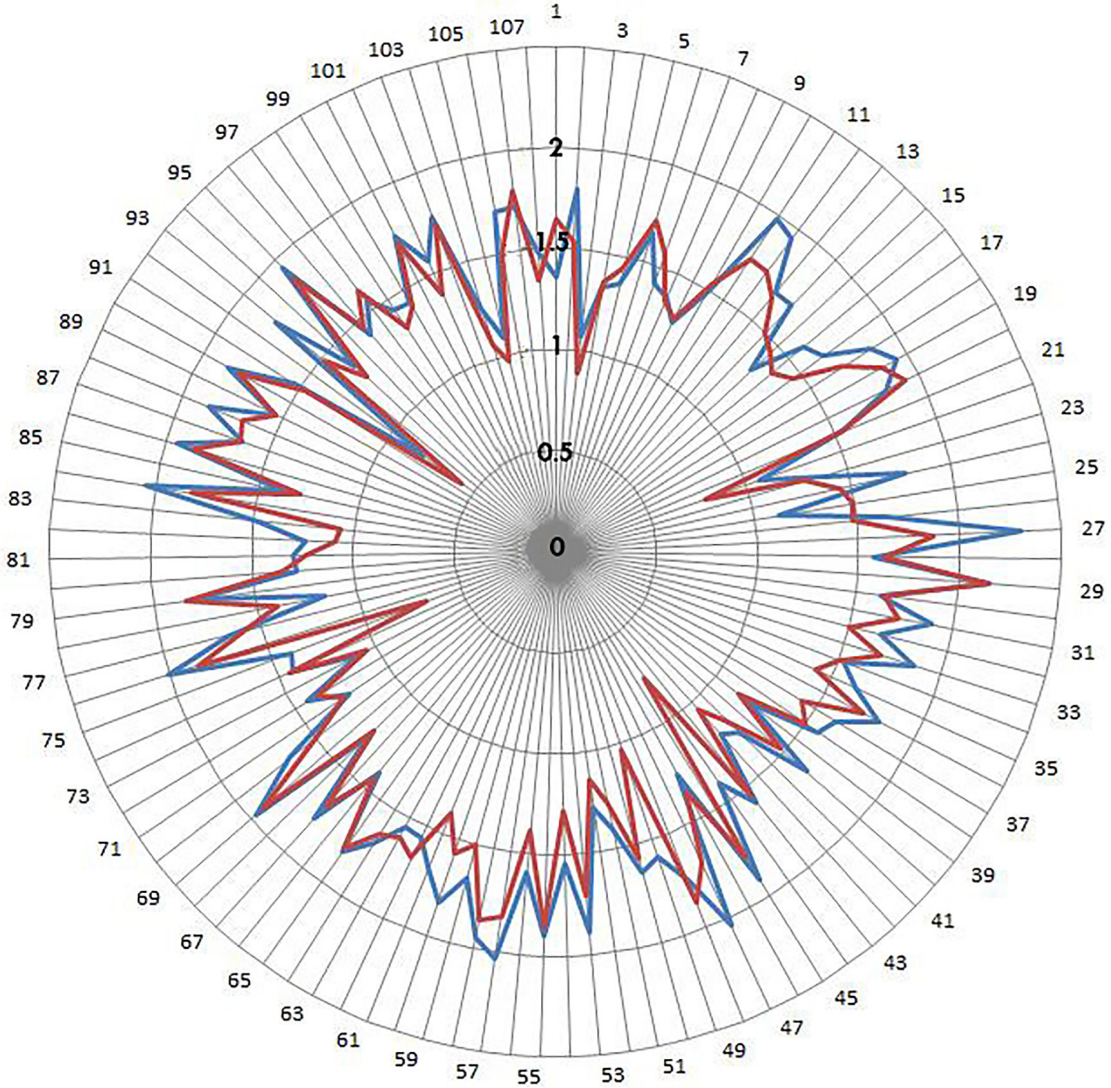
Radar chart for Delphi survey round 2 and round 3 responses. Distribution of the standard deviations for each of the 107 Delphi survey questions (from 1 to 107, in the clockwise direction) in round 2 (*blue line*) and round 3 (*red line*). Note that the distribution of the standard deviations across the 107 survey questions is dampened in round 3 (*red line*) as anticipated in Delphi surveys.

The threshold for “consensus” was established *a priori*. If at least 75% of the experts rated a question as one of the top two (a score of six or seven on the 7-point Likert scale) *or* bottom two (a score of one or two) scores, the survey question was considered to have achieved consensus. Conversely, the respondents were deemed to have “dissensus” on a survey question if the bottom 3 points as a group (a score of one, two or three) *and* the top 3 points as a group (a score of five, six or seven) *each* gathered at least 33% of ratings. The respondents’ ratings of the questions between the second and the third round were compared visually by plotting the standard deviations for each of the survey question (**Fig 4**).

## Results and Discussion

To the best of our knowledge, the present report is the first Delphi study on the emergence of metagenomics technology that is anticipated to transform water safety testing in public health practice and ecosystem research. The description of the 24 respondents who completed the Delphi survey is displayed in **Table 2**. This is consistent with and above the sample size typically employed in Delphi surveys [35, 36]. As a general concept, the aim of the Delphi surveys is not statistical hypothesis testing but to obtain an open evaluation of viewpoints from experts so as to inform future science and technology policy and new practices of doing science.

**Table 2:** Description of the Respondents who Completed the Delphi Survey (N = 24).

**Sex**	
Female	8
Male	16
**Median Age:** Median year (quartile 1—quartile 3)	51 (43–58)
**Professional experience:** Median year (quartile 1—quartile 3)	13 (10–20)
**Sector of employment** ^**a**^	
Government	16
University	7
Industry	3
**Highest level of education** ^**b**^	
PhD	14
Master’s	4
Bachelor’s	5

**a:** Two respondents were counted twice as they self-identified as being employed both by government and by universities.

**b:** One respondent declined to provide this demographic information.

### Priority Evidentiary Criteria to Adopt Metagenomics Tests

We identified the top 10 priority evidentiary criteria perceived to be important by the respondents to guide future decisions on adoption of metagenomics tests for water safety (**Table 3**). The ability of metagenomic tests to distinguish between harmful and benign organisms in water, a test’s analytical and clinical sensitivity, reproducibility from lab-to-lab and equipment-to-equipment, were among the top three noted criteria. Public and ecosystem health utility of a test was deemed to be also important: e.g., the extent to which a given metagenomics profile contributes to risk associated with the presence of a waterborne pathogen, and whether the test can be calibrated to health related endpoints (e.g., illness, mortality or reduction in boil water advisories) (**Table 3**). This suggests that the experts are cognizant of the need to demonstrate a test’s analytical and clinical sensitivity/specificity–i.e., whether the metagenomics profiles can be detected in a given water sample, and whether they are associated with presence of pathogen(s)–before a test’s broader utilities are considered in due course.

**Table 3:** Top 10 Decision Criteria to Adopt Metagenomic Testing for Water Safety.

Rank	Item
1	Ability to distinguish between harmful & benign organisms
2/3*	Test’s sensitivity (analytical and clinical)
2/3*	Test’s reproducibility/repeatability lab-to-lab and equipment-to-equipment
4	Metagenomic testing is at least as affordable as current water monitoring technology
5	Test’s specificity (analytical and clinical)
6	Definition of safe water
7	The extent to which a given metagenomics profile contributes to risk associated with the presence of a pathogen
8/9*	Consider whether presence of pathogens in water is directly correlated to water-borne disease
8/9*	Can the test be calibrated to public health endpoints (e.g., illness or mortality)?
10	A metagenomics test must be sufficiently robust to be applied across a variety of water samples

*tied.

Respondents had consensus that metagenomics tests will offer “a better understanding of pathogens and of the risks associated with their presence in the water” (**Table 4**). Yet, as discussed below, experts also note that a metagenomics profile will more likely serve as ancillary criterion in decision-making for water safety, rather than a standalone decision instrument. We should mention that such perceptions tend to be dynamic constructs, particularly for rapidly emerging technologies and innovations. Conceivably, metagenomics technology may potentially be “upgraded” in the future to a core decision-making test status by water safety experts as its evidence base accumulates. In the meantime, however, because perceptions on what is most essential evidence serve a critical gatekeeper function to transition (or not) emerging biotechnologies to practice, the top 10 decision criteria perceived to be important by experts can help prioritize the current research agenda in the field of metagenomics and successfully navigate the boundary between metagenomics laboratory discoveries and their adoption as new water safety tests (**Table 3**).

**Table 4:** Ranking of the Issues with CONSENSUS Among Experts in Regards to Metagenomic Tests for Water Safety.

Rank	Issue	Type of Scale	Consensus Direction	Level of Consensus (%)
1	The fact that metagenomic testing for water safety is currently available as a commercialized product/service is sufficient reason for its acceptance for use by policy-makers.	Agreement/ Disagreement	*Disagreed*	*96*
2	The general public understands the technical issues related to the subject of water testing.	Agreement/ Disagreement	*Disagreed*	*96*
3	Detailed evidence on the benefits of the technology is prepared before the publics are engaged.	Desirability	*Desirable*	*91*
4	Metagenomic test results will be used by advocacy groups seeking to advance their political interests.	Agreement/ Disagreement	*Agreed*	*91*
5/6	Government officials, e.g., health authority officials, are a public relevant to the issue of public engagement on metagenomic testing for water safety.	Agreement/ Disagreement	*Agreed*	*83*
5/6*	Scientists are a public relevant to the issue of public engagement on metagenomic testing for water safety.	Agreement/ Disagreement	*Agreed*	*83*
7	Academia is a public relevant to the issue of public engagement on metagenomic testing for water safety.	Agreement/ Disagreement	*Agreed*	*78*
8/9	Metagenomic testing will lead to a better understanding of pathogens and of the risks associated with their presence in the water.	Agreement/ Disagreement	*Agreed*	*76*
8/9*	Standardized acceptable methods and accredited laboratories will be adopted for metagenomic testing.	Desirability	*Desirable*	*76*
10	Marker-gene assessment needs to be combined with other evidence in order to provide a more authoritative prediction of water safety.	Agreement/ Disagreement	*Agreed*	*75*
11	Metagenomic testing will be used in source water assessments in relation to land/watershed use and possible risk factors related thereto.	Desirability	*Desirable*	*75*

*Tied ranking

In all, scientists and policymakers are cognizant that metagenomics biotechnology might play an important role in public health practice if priority evidentiary requirements are met.

### Consensus and Dissensus on Metagenomics Tests

Out of the 107 questions surveyed, we observed consensus on 11, and dissensus on 12 items (**Tables 4 and 5**, respectively). Consistent with experts’ tendency to dampen their personal opinion deviations from the group means, the expressed perceptions of the experts displayed lesser variability in round 3 compared to round 2 of the Delphi survey (**Fig 4**). Among the issues with consensus, the commercial availability of metagenomics tests/services, alone, was considered an insufficient reason to adopt them for water safety testing. Experts had consensus that metagenomics biomarker data need to be combined with other evidence in order to provide a more authoritative prediction of water safety (**Table 4**). We observed that 71% of the respondents gave a strong affirmative response (either 6 or 7 on the 7-point Likert scale) to the question “metagenomic testing will be a supplementary method utilized in testing for organic contamination in water, as opposed to becoming the exclusive technology used”.

**Table 5:** Ranking of the Issues with DISSENSUS Among Experts in Regards to Metagenomic Tests for Water Safety.

Rank	Item	Type of Scale	COLUMN A % Agreed or Feasible	COLUMN B % Disagreed or Unfeasible	Level of *DISSENSUS* (%) (Columns A + B)
1	People with little knowledge of and/or experience in water safety issues and assessment procedures are a public relevant to the issue of public engagement on metagenomic testing for water safety.	Agreement/ Disagreement	39.1	60.9	*100*
2	Metagenomic testing for water safety will lead to more confusion regarding the assessment of public health issues related to water safety.	Agreement/ Disagreement	61.9	33.3	*95*.*2*
3	Metagenomic profiles can be reliably used to contribute to the analysis of risk associated with the presence of a pathogen.	Agreement/ Disagreement	57.9	36.8	*94*.*7*
4	Metagenomic testing will justify replacing “indicator” organisms with those organisms that have been shown to be pathogenic.	Agreement/ Disagreement	33.3	57.1	*90*.*4*
5	Detailed evidence on the benefits of the technology is prepared before the publics are engaged.	Feasibility	57.1	33.3	*90*.*4*
6	A database of genomic profiles of pathogens potentially inhabiting a given watershed is a prerequisite to the use of metagenomic testing for determining the safety of the watershed.	Agreement/ Disagreement	36.8	52.6	*89*.*4*
7	Lawyers are a public relevant to the issue of public engagement on metagenomic testing for water safety.	Agreement/ Disagreement	52.2	34.8	*87*.*0*
8	There is evidence to indicate that the current water testing regime is lacking in terms of consumer protection and water safety.	Agreement/ Disagreement	47.8	39.1	*86*.*9*
9	Metagenomic testing will play a role in eliminating unnecessary boil water advisories.	Agreement/ Disagreement	40.9	45.5	*86*.*4*
10	The publics will be engaged in the process of the application of metagenomic testing for water safety.	Feasibility	39.1	43.5	*82*.*6*
11	Metagenomic testing will be highly *specific* in detecting pathogen phenotypes in the water, i.e. will avoid type I errors in *clinical validity* (false positives).	Agreement/ Disagreement	33.3	38.9	*72*.*2*	
12	Metagenomic testing will be highly *specific* in detecting the genomic profiles in the water, i.e. will avoid type I errors in *analytical validity* (false positives).	Agreement/ Disagreement	33.3	33.3	*66*.*7*	

*Dissensus* is observed for a survey question if on the 7-point Likert scale, the bottom 3 points as a cluster (a score of one, two or three), *and* the top 3 points as a cluster (a score of five, six or seven), *each* gathered at least 33% of ratings. *Dissensus* is the sum of the ratings gathered from the top and bottom 3 point clusters on the 7-point Likert scale (Columns A + B above).

Consistent with a perceived ancillary decision-making role for metagenomics tests, the respondents’ views were polarized on the extent to which a metagenomics test result will be the primary criterion for water safety. For example, they had dissensus on the question whether “metagenomic profiles can be reliably used to contribute to the analysis of risk associated with the presence of a pathogen” (**Table 5**). Similarly, the respondents displayed dissensus on whether or not the “metagenomic testing will play a role in eliminating unnecessary boil water advisories” (**Table 5**).

While analytical and clinical validity of a metagenomics test were considered among the top 10 priority evidence for adoption in water safety, experts were polarized on whether specificity of a test can be achieved to avoid false positives (**Table 5**, *dissensus items 11 and 12*). Moreover, the experts do not appear to broadly converge on the idea that the current water testing regime is lacking in terms of consumer protection and water safety (**Table 5**, *dissensus item 8*).

### Perceived Role and Timing of Public Engagement

#### Who should generate the evidence for adoption of metagenomics tests

In terms of the “co-production” of evidentiary base on emerging technologies and innovations, as evidenced by new scientific practices such as citizen science well-known in conservation science and ecology, there is a shift towards working with a broader range of innovation actors outside the laboratory including the citizens, nongovernmental organizations, patient advocacy groups, among others. While the science and policy experts in the present Delphi study agreed with consensus that governments, scientists and academics are legitimately “a public relevant to the issue of public engagement on metagenomic testing for water safety”, interestingly, they did not display similar consensus on the need for engagement with the general public.

In these findings, it is striking that the experts do not appear to see a major proactive need for engagement with the general public, a key user community for the anticipated metagenomics tests, beyond a passive downstream role for adoption of a technology. For example, science and policy experts in the present study have consensus that “detailed evidence on the benefits of the technology is prepared before the publics are engaged” (**Table 5**), rather than a substantive and democratic co-productionist role for public engagement at an upstream *design* and *discovery* stage of metagenomics tests.

These perceptions of the experts appear to stem from a linear and narrow innovation model focussing primarily on the classic “*push factors*” (scientists and laboratory actors) instead of a bi-directional model of innovation and knowledge co-production where both technology inventors/designers and user communities such as the general public (*pull factors*) are engaged in collective innovation. Notably, such perceptions run against the latest scholarship from social studies of science and technology [24, 29] and the emerging field of “citizen science” where user communities, and publics more generally, are understood to be capable of making productive contributions to design, funding and/or implementation of scientific projects [29, 39].

### Opportunities for Policymaking on Emerging Water Safety Tests

In Delphi studies, issues with consensus may point to socio-technical areas where contestation or conflict among the innovation actors may be less likely. But the reverse side of the consensus medallion is also noteworthy. When there is consensus on a given subject, it may mean the experts are already “entrenched” firmly in their opinions and are unlikely to change their stances easily; they might be resistant to guidance by new insights or innovation policies on that subject matter. Hence, issues where there is no consensus at all are worthy of careful reconsideration for future policy design because such topics without a consensus might actually be the real-life *actionable* target issues where “change is still possible” by new policies. Such a focus on topics with no consensus would serve the metagenomics science, policy and user communities well by helping steer the attendant innovation trajectory from lab to society. We therefore focus below on “open” issues where we did not observe a consensus or dissensus.

Chief among these open issues with no consensus is that the experts do not appear to have yet formed a firm opinion on the breadth of plausible applications for metagenomics tests. Indeed, there was no consensus, nor dissensus, on the latter subject that was articulated in the Delphi survey with the query “metagenomic tests will be deployed at many more locations than current tests are” (**S1 Text**). Nor did they display a dissensus/consensus for tailoring the metagenomics tests for different application contexts such as watershed versus tap water (Query: “Metagenomic testing will be tailored to specific monitoring settings”).

Metagenomics testing, in theory, can be utilized not only for assessing water safety or baseline contamination but also the extent of *change* in water organic contamination after treatment with candidate interventions aimed at improving ecosystem health. The experts did not show dissensus/consensus on this subject matter either, suggesting that the metagenomics innovation trajectory is “wide open” in these particular dimensions to be shaped by future technology policies.

As an ancillary observation, we report that more than half of the experts (59%) opined that if metagenomic testing were to be used for water safety, this would materialize in 4 to 9 years.

It is noteworthy that the Delphi method is a bottom-up procedure with survey questions being emergent from the field (proposed largely by the respondent experts). Thus, Delphi as a form of social science research is distinct from investigator-planned top-down questionnaires that may be detached from the field realities at the design stage since how one asks the questions in part determines the answers [40–42]. In this sense, the Delphi method is aligned directly with the values and perceptions of the innovation actors in the field starting with the survey design and the answers obtained. A Delphi survey is also different from interviews with field respondents and do not always permit further questions on the mechanisms of consensus or dissensus. In our study, the respondents noted that metagenomics tests would provide crucial new information but that they may not necessarily replace the current water safety tests. Mechanisms of these perceptions warrant further social science research by complementary methodologies, for example, using interviews and ethnography research.

We shall note that perceptions and values of individuals and professional communities of practice do change over time with new socio-technical developments such as personal genomics, direct-to-consumer sale of new diagnostics that bypass public health systems, new legal reforms pertaining to ecosystem health and safety, not to forget amendments to future innovation policies [43–45]. It would serve the public health community well to conduct repeated Delphi surveys; so dynamic and real-time monitoring of both emerging technologies and the values of the attendant innovation actors can be mapped continuously [46, 47]. Such “socio-technical” evidence is essential for responsible governance of novel technologies such as metagenomics.

Finally, we caution the reader that the present qualitative observations specifically, and the Delphi surveys more broadly, are *not* meant to be a hypothesis test on metagenomics technology futures. Instead, the findings provide anticipatory knowledge that can guide future innovation policy in regards to novel water safety tests emerging from metagenomics applications. We suggest future research in the field may take on issues that relate, for example, decision-makers’ attitudes to their levels of expertise and whether or not they had used metagenomics technology in the past. Notably, however, the experts who participated in the present survey had a median 13 years of professional experience in science and/or public health policy and majority had a PhD degree (**Table 2**). Because our aim was not quantitative hypothesis testing of the putative determinants of inter-individual variability in experts’ perceptions on metagenomics, these types of issues offer opportunities for future social science research.

## Conclusions

Metagenomics technology, owing to its promise to detect the waterborne pathogens directly, is poised to be a potential game changer for water safety testing. To the best of our knowledge, there is no published empirical study of science and policymaker experts’ attitudes towards the priority evidentiary criteria to transition a laboratory metagenomics discovery to water safety tests in practice. Scientific evidence is situated as a precursor filter between lab and societal applications of emerging technologies such as metagenomics (**Fig 1**). Surprisingly, attitudes of experts towards the types and extent of scientific evidence on metagenomics have not been studied to date. The present study thus makes an important contribution to build anticipatory capacity on metagenomics and ecosystem health innovations by reporting the top 10 decision criteria deemed to be important by science and policy experts for adoption of metagenomics technology in practice (**Table 3**). Additionally, the issues on which there is consensus and dissensus are reported, together with the contexts in which the experts have not yet formed a firm opinion. These observations point at strategic contexts where policy interventions may be most effective, and situations where conflict and synergies among innovation actors might be anticipated and managed in advance, as discussed above.

Experts agree that metagenomic testing will provide valuable new information on organic water contamination but there is dissensus on whether or not metagenomics will replace the current water safety testing methods or reduce the boil water advisories. Experts view the general publics relevant in a “downstream capacity”: to adopt metagenomics tests in a linear innovation model from lab-to-society, rather than a substantive co-productionist role at the “upstream” scientific design stage of metagenomics tests.

Taken together, these findings offer timely anticipatory knowledge on the metagenomics innovation trajectory; experts appear to perceive the latter as an incremental linear innovation instead of a game changing disruptive biotechnology innovation. A closer engagement among experts and publics should, however, bring about a deeper appreciation of the broader range of metagenomics applications and the ways in which technology inventors and user communities might engage for co-production of metagenomics innovations.

## Strategic Outlook for Anticipatory Technology Governance

Innovations, by definition, are often unprecedented nor can we predict their trajectory entirely. But we can give direction to innovations and emerging technologies so they steer towards outcomes that are grounded in societal values and priorities. One way to achieve this objective is to engage with emerging technologies such as metagenomics from their outset at an “upstream” discovery/design stage before the opinions on the innovation trajectory are “locked in”, and while it is still possible to shape the innovation trajectory. Small changes by early policy interventions on new technologies accrue over time as a technology differentiates and diffuses into new applications.

As a concrete analogy, innovations and emerging technologies are akin to pluripotent stem cells that can differentiate into various tissue types, given sufficient context and direction. Similarly, technology foresight data can provide direction and allow anticipatory governance of emerging technologies as they differentiate into various applications in society. In our case, we wish to note that the present study, by virtue of its focus on how evidence on metagenomics technology is currently being perceived by science and policy experts, offers strategic foresight and future policy guidance on metagenomics innovations. Our study focus on potential social shaping of scientific evidence by experts’ values and perceptions is particularly novel from a social science context as scientific evidence, historically, has been considered to be invariably objective and not subject to influences by scientists’ or policymakers’ value systems. The findings thus collectively offer new insights at the intersection of metagenomics technology, society and innovation policy. The data also suggest ways forward to inform future anticipatory ethics and technology governance frameworks whereby both innovation actors and innovation narrators work in tandem to examine and steer emerging biotechnologies such as metagenomics to responsible innovation [48–55].

As environmental regulatory agencies struggle to develop novel indicators of water pollutants and ecosystem health, metagenomics tests warrant further consideration and evidence-based assessment for their utility to impact the public health related endpoints such as boil water advisories. Our observations made here suggest the ways in which metagenomics evidence can be subject to influences by varied perceptions of scientists and policymakers, and to the best of our knowledge, is the first study that examined the potential social construction of scientific evidence on metagenomics driven diagnostics. Because evidence is a precursor determinant of the user uptake of innovative products (**Fig 1**), we need to remain vigilant for potentials in regards to social construction of scientific evidence by innovation actors, and the ways in which evidence shapes, and is shaped by decision makers’ perceptions on novel diagnostics for ecosystem health.

Finally, steering or managing innovations in a sustainable and responsible manner is not so different than a complex aerodynamic system like an airplane that requires governance instruments for both *acceleration* and *deceleration*. Delphi surveys and anticipatory knowledge generated by social science and humanities research offer exciting opportunities to govern innovations *symmetrically*: by identifying areas where acceleration (e.g., consensus areas) and deceleration/reconsideration (e.g., dissensus areas) of the innovation trajectory are warranted.

Looking ahead, we cannot help wonder the uptake of such anticipatory knowledge when, for example, a momentary deceleration of the scientific discovery engine may be prudent for long-term product uptake or innovation ecosystem and multilateral stakeholder sustainability [51, 52]. How might stakeholders, funding agencies, large consortia ethicists and scientists, governments and other innovation actors with existing political, career and economic commitments view such foresight data and social science recommendations for deceleration and acceleration of emerging technology applications?

Our answer is that anticipatory governance of emerging technologies could not be more important, valuable and timely, for sustainability of ecosystems and prosperity of global society. After all, any sensible innovation actor, be they governments, scientists, consortia ethicists or funding agencies would like to know the future(s) of the innovation trajectory in advance. Indeed, the fast approaching special meeting at the United Nations in September 2015 is dedicated to approving the SDGs for the 2016–2030 term. The SDGs are envisioned as successors to the Millennium Development Goals (MDGs) that were agreed upon in 2000 and expiring in 2015. As the world’s safe water supplies continue to be threatened by organic and inorganic contamination, we ought to think beyond our immediate self-interests as persons, communities or governments [26, 28, 51, 56–67]. Hence, we call for continued, rigorous and *independent* trustworthy social science and humanities research on new *nested* governance approaches (e.g., ethics as well as ethics-of-ethics) for emerging diagnostic technologies such as metagenomics that have vast importance for both ecosystems and human health [51, 55–57, 59, 60].

## Supporting Information

S1 Text The Delphi survey questions. (DOCX)
