# Supplementary material for: A Delphi Technology Foresight Study: Mapping Social Construction of Scientific Evidence on Metagenomics Tests for Water Safety
Source: PLoS One. 2015 Jun 11;10(6):e0129706. doi: 10.1371/journal.pone.0129706 (PMC4465892; doi:10.1371/journal.pone.0129706)
Supplement: S1 Text — (DOCX) [file pone.0129706.s001.docx]

**S1 Text. Delphi Survey of Experts’ Views on Metagenomics Tests for Water Safety**

We request that you rate each survey question on the 7-point Likert scale* provided under each question. Note that some questions are rated on more than one scale (e.g., desirability and feasibility) each of which counts as an itemized response to that question.

The names of each applicable scale are provided within parentheses at the end of each survey question.

*For the sake of brevity as a supplemental table, the scales are removed from the table but example scales are provided below.

***Agreement Scale***

__1 __2 __3 __4 __5 __6 __7
 Do not agree Agree fully

at all

***Desirability Scale***

__1 __2 __3 __4 __5 __6 __7
 Not desirable Most desirable

at all

***Feasibility Scale***

__1 __2 __3 __4 __5 __6 __7
 Not feasible Definitely feasible

at all

**A. Introductory Questions**

1. New tests for water safety are needed. (Scale – Agreement)

2. There is evidence to indicate that the current water testing regime is lacking in terms of consumer protection and water safety. (Scale – Agreement)

3. The fact that metagenomic testing for water safety is currently available as a commercialized product/service is sufficient reason for its acceptance for use by policy-makers. (Scale – Agreement)

4. Metagenomic testing will be a supplementary method utilized in testing for organic contamination in water, as opposed to becoming the exclusive technology used. (Scale – Agreement)

5. Metagenomic testing will provide crucial new information that current tests cannot. (Scale – Agreement)

6. Metagenomic testing will be used to assess the quality of raw surface water sources for drinking water. (Scale – Agreement)

7. Metagenomic testing will be used to assess the quality of treated water. (Scale – Agreement)

**B. Evidentiary Requirements for Adoption of Metagenomics Tests**

**Analytical Validity**

8. Metagenomic testing and the related analyses will be able to accurately identify the genomic profiles in the water. (Scale – Agreement)

9. Metagenomic testing will be highly *sensitive* in detecting the genomic profiles in the water, i.e., will avoid type II errors in analytical validity (false negatives). (Scale – Agreement)

10. Metagenomic testing will be highly *specific* in detecting the genomic profiles in the water, i.e., will avoid type I errors in analytical validity (false positives). (Scale – Agreement)

11. A database of genomic profiles of pathogens potentially inhabiting a given watershed is a prerequisite to the use of metagenomic testing for determining the safety of the watershed. (Scale – Agreement)

12. The establishment of baseline metagenomic profiles of a given watershed is a prerequisite to the use of metagenomic testing for determining its safety. (Scale – Agreement)

13. Metagenomic test analytical validity will be repeatable, i.e., consistently reproducible with the same or additional equipment in the same or additional laboratories. (Scale – Agreement)

14. Water samples required for reliable extraction, purification, and detection of contaminant microorganism DNA are easily obtainable. (Scale – Agreement)

**Clinical/Ecosystem Validity**

15. Metagenomic testing will be able to predict the presence of a specific contaminant microorganism based on the metagenomic profile of the source. (Scale – Agreement)

16. Metagenomic testing will be able to accurately and quantitatively measure the concentration of the specific pathogens of concern in the water. (Scale – Agreement)

17. Metagenomic testing will be highly *sensitive* in detecting pathogen phenotypes in the water, i.e., will avoid type II errors in clinical validity (false negatives). (Scale – Agreement)

18. Metagenomic testing will be highly *specific* in detecting pathogen phenotypes in the water, i.e., will avoid type I errors in clinical validity (false positives). (Scale – Agreement)

19. Metagenomic testing will be able to accurately distinguish between harmful and benign pathogens. (Scale – Agreement)

**Utility**

20. Metagenomic profiles can be reliably used to contribute to the analysis of risk associated with the presence of a pathogen. (Scale – Agreement)

21. Metagenomic testing needs to be built on thorough validation of particular genomic markers with consideration of both analytical and clinical/ecosystem validity. (Scale – Agreement)

22, 23. Metagenomic testing will be calibrated to public health endpoints (e.g., illness, mortality). (Scales – Desirability, Feasibility)

24. Presence of pathogens in water is directly correlated to water-borne disease. (Scale – Agreement)

25, 26. Metagenomic test outcomes will be equated to standards for water safety indicators currently used by public health officials. (Scales – Desirability, Feasibility)

27. New standards/guidelines on what is safe water are required before a metagenomic test’s ability to predict water contamination can be assessed. (Scale – Agreement)

**C. Other Test Attributes**

28. Obtaining metagenomic test results will be at least as timely as existing tests. (Scale – Agreement)

29. On the following scale, please indicate how long you think it will take on average to obtain results using metagenomic testing, excluding the time required for sample collection. (Scale – 1 min, 30 min, 2 hrs, 12hrs, 24hrs, 2days, ≥ 4days)

30, 31. Metagenomic testing will be adapted for use with an online platform, e.g., an online bioinformatics platform, online repository/database, etc. (Scales – Desirability, Feasibility)

32, 33. Metagenomic tests will be deployed at many more locations than current tests are. (Scales – Desirability, Feasibility)

34, 35. Metagenomic testing will be tailored to specific monitoring settings. (Scales – Desirability, Feasibility)

36. Metagenomic testing instrumentation will require more maintenance than current test instrumentation. (Scale – Agreement)

37. Metagenomic testing will be at least as affordable as current water monitoring technology. (Scale – Agreement)

38. Metagenomic testing will be at least as easy to perform as current tests. (Scale – Agreement)

39, 40. Metagenomic testing will reliably use multiplexing strategy to sequence many samples at once. (Scales – Desirability, Feasibility)

41, 42. Metagenomic testing will be primarily taxonomic in nature. (Scales – Desirability, Feasibility)

43. Taxonomic precision is sufficient for metagenomic testing. (Scale – Agreement)

44. Marker-gene assessment needs to be combined with other evidence in order to provide a more authoritative prediction of water safety. (Scale – Agreement)

45, 46. Environmental shotgun sampling will be used to profile a sample of all the genetic material present at a given place and time. (Scales – Desirability, Feasibility)

**Interpretation**

47. Metagenomic test results will be at least as easy to interpret by the test’s operator as the results of current tests. (Scale – Agreement)

48, 49. All metagenomics related algorithms will be understandable by users, i.e., there will be no “black box” bioinformatics. (Scales – Desirability, Feasibility)

50. The amount of information derived from the analysis of metagenomic test results will be adequate for the attendant decision-making process. (Scale – Agreement)

51. Current data analysis techniques are adequate for dealing with the results of metagenomic profiling of water environments. (Scale – Agreement)

52. There exists significant potential for greater misrepresentation of metagenomic test results by selective presentation of “key” areas of the dataset. (Scale – Agreement)

53. Metagenomic testing will be definitive, i.e., no confirmatory testing for organic contamination will be needed. (Scale – Agreement)

54. In how many years, in your opinion, will metagenomic testing be ready to be used for water safety, if at all? (Scale – Now; 1-3 years; 4-6 years; 7-9 years; 10-12 years; 13+ years; Never)

**D. Consequences of Metagenomics Tests**

**Identification of pathogens**

55. Metagenomic testing will help trace the source of a specific contamination event. (Scale – Agreement)

56, 57. Metagenomic test results will be admitted as legal evidence in determining liability for contamination. (Scales – Desirability, Feasibility)

58. Metagenomic testing will contribute to reducing public exposure to pathogens. (Scale – Agreement)

**Understanding**

59. Metagenomic testing will lead to a better understanding of pathogens and of the risks associated with their presence in the water. (Scale – Agreement)

60. Metagenomic testing for water safety will lead to more confusion regarding the assessment of public health issues related to water safety. (Scale – Agreement)

61. Metagenomic profiling of contaminants and potential source communities will allow for geospatially-enabled databases to be built that could greatly enhance current efforts at pathogen identification and prevention of watershed contamination. (Scale – Agreement)

**Treatment, Standards & Regulations**

62, 63. Metagenomic testing will result in the introduction of new water treatment regulations. (Scales – Desirability, Feasibility)

64. From 0 to 100%, in your opinion, how much of the water treatment conducted as a result of metagenomic testing will carry unjustified risks (public health, economic, etc.)? (Scale – 0%; 1-20%; 21-40%; 41-60%; 61-80%; 81-99%; 100%)

65. Metagenomic testing will justify replacing “indicator” organisms with those organisms that have been shown to be pathogenic. (Scale – Agreement)

66, 67. Metagenomic testing will be used to measure the effectiveness of different water treatment systems. (Scales – Desirability, Feasibility)

68, 69. Standardization of acceptable methods and accredited laboratories will be adopted for metagenomic testing. (Scales – Desirability, Feasibility)

**Economic and political consequences**

70. Metagenomic testing will lead to more effective management of resources that address risks related to water safety. (Scale – Agreement)

71, 72. Metagenomic testing will be used in source water assessments in relation to land/watershed use and possible risk factors related thereto. (Scales – Desirability, Feasibility)

73, 74. Metagenomic testing will assist regulatory agencies with the monitoring of transboundary waters. (Scales – Desirability, Feasibility)

75. Metagenomic test results will be used by advocacy groups seeking to advance their political interests. (Scale – Agreement)

76. Metagenomic testing will play a role in eliminating unnecessary boil water advisories. (Scale – Agreement)

77. Metagenomic testing will lead to increased public confidence in the safety of the water. (Scale – Agreement)

78, 79. Metagenomic test results will lead to authorities targeting specific contaminant sources. (Scales – Desirability, Feasibility)

**E. Public Engagement**

Below, we use the word “publics” in plural because we talk of a number of distinct publics, whether they are laypeople or groups having a specific relationship with the technology. Speaking of “the general public”, on the other hand, we refer to the society at large regardless of membership to any particular group.

80. Government officials, e.g., health authority officials, are a public relevant to the issue of public engagement on metagenomic testing for water safety. (Scale – Agreement)

81. Academia is a public relevant to the issue of public engagement on metagenomic testing for water safety. (Scale – Agreement)

82. Scientists are a public relevant to the issue of public engagement on metagenomic testing for water safety. (Scale – Agreement)

83. Water treatment operators and staff are a public relevant to the issue of public engagement on metagenomic testing for water safety. (Scale – Agreement)

84. People with little knowledge of and/or experience in water safety issues and assessment procedures are a public relevant to the issue of public engagement on metagenomic testing for water safety. (Scale – Agreement)

85. Laypeople with significant knowledge of and/or experience in water safety issues and assessment procedures are a public relevant to the issue of public engagement on metagenomic testing for water safety. (Scale – Agreement)

86. Lawyers are a public relevant to the issue of public engagement on metagenomic testing for water safety. (Scale – Agreement)

87. Doctors are a public relevant to the issue of public engagement on metagenomic testing for water safety. (Scale – Agreement)

88. People with particular sensitivities or vulnerabilities to water contamination are a public relevant to the issue of public engagement on metagenomic testing for water safety. (Scale – Agreement)

89. Social activist groups and NGOs are a public relevant to the issue of public engagement on metagenomic testing for water safety. (Scale – Agreement)

90, 91. The publics will be engaged in the process of the application of metagenomic testing for water safety. (Scales – Desirability, Feasibility)

92. The general public understands the technical issues related to the subject of water testing. (Scale – Agreement)

93. The general public is interested in the subject of water safety technology. (Scale – Agreement)

94. The general public is biased against metagenomic technology. (Scale – Agreement)

95. The general public will expect too much from metagenomic testing. (Scale – Agreement)

96, 97. Science and/or technology experts will educate the publics about metagenomic testing. (Scales – Desirability, Feasibility)

98, 99. Public upstream engagement* will happen in regards to metagenomic testing. (Scales – Desirability, Feasibility)

**Public upstream engagement is public dialogue and deliberation about technologies at the early stages of their R&D process and in advance of significant application with the potential to influence technology trajectories.*

100, 101. The publics will be engaged in science, e.g., by collecting and sharing data relevant to water testing. (Scales – Desirability, Feasibility)

102, 103. The publics will be engaged as early as possible in the processes of development and acceptance of metagenomic testing technology. (Scales – Desirability, Feasibility)

104, 105. Detailed evidence on the benefits of the technology is prepared before the publics are engaged. (Scales – Desirability, Feasibility)

106. Public engagement will distract from a proper decision-making process in water safety. (Scale – Agreement)

107. Information in regards to metagenomic testing of water presented by the media to the publics may be trusted to be fair. (Scale – Agreement)

**F. Setting the Priority Issues for Decisions on Adoption of Metagenomics Tests**

Please choose and rank what issues you consider to be the top 10 priorities to be considered in the decision whether or not metagenomic testing should be widely adopted for water safety. Please choose from the list provided below, which is taken or emergent from the previous questions.

1. A metagenomics test must be sufficiently robust to be applied across a variety of water samples
2. Ability to deploy the test at several locations
3. Ability to distinguish between harmful & benign organisms
4. Ability to measure the concentration of a pathogen
5. Ability to relate to standards for current water safety indicators
6. Ability to tailor test to monitoring settings
7. Amount of information derived from metagenomics profiling
8. Consider whether presence of pathogens in water is directly correlated to water-borne disease
9. Definition of safe water
10. Definitiveness of the test, i.e., need for confirmatory testing
11. Ease of interpreting test results
12. Ease of performing the test
13. Establishment of baseline conditions
14. Existence of a “critical limit” of a pathogen in causing disease
15. Existence of a database of metagenomics profiles
16. Existence of “black box” bioinformatics
17. Identification of the metagenomics profile
18. Instrumentation maintenance
19. Possibility of calibration to public health endpoints (e.g., illness or mortality)
20. Potential for greater misrepresentation of metagenomics test results
21. Reliability of DNA detection from water
22. Sufficiency of marker-gene assessment
23. Sufficiency of taxonomic precision
24. Test’s analytical and clinical sensitivity
25. Test’s analytical and clinical specificity
26. Test’s cost
27. Test’s reproducibility/repeatability lab-to-lab and equipment-to-equipment
28. Test’s timeliness/turn around times
29. The extent of validation of genetic markers
30. The extent to which a given metagenomics profile contributes to risk associated with the presence of a pathogen.

**Please give your ranking below:**

1.

2.

3.

4.

5.

6.

7.

8.

9.

10.

**End of Questionnaire**

**Thank you**
